# Persistent lactation in bilateral breast implant augmentation: A case report and review of the literature

**DOI:** 10.1016/j.jpra.2024.02.006

**Published:** 2024-02-15

**Authors:** Mary Goble, Nicholas Cereceda-Monteoliva, Naveen Cavale

**Affiliations:** a King's College Hospital, London, United Kingdom; b Guy's and St Thomas’ Hospital Trust, London, United Kingdom

**Keywords:** Aesthetic breast surgery, Breast augmentation, Breast reconstruction, Persistent lactation, Case report, Literature review

## Abstract

**Background:**

Persistent lactation, or galactorrhoea, is a common problem which is infrequently seen in the setting of aesthetic surgery. Increasing frequency of aesthetic breast surgery such as breast augmentation suggests a need for improved understanding of the effect of galactorrhoea on surgical outcomes.

**Case Report:**

A 34-year-old patient underwent day-case bilateral breast reduction/mastopexy combined with sub-muscular implant augmentation, abdominoplasty and bilateral liposuction to the flanks. She reported to have stopped breastfeeding more than 6 months prior. Intraoperatively, the breast tissue was noted to be lactating. The procedure was completed as planned and a routine postoperative plan was followed including oral antibiotics, analgesia and compression garments. The patient was discharged, however reattended on postoperative day 10 with breast pain and fevers. She was treated for right breast surgical site infection and required washout and implant removal. She was referred to Endocrinology for treatment of galactorrhoea with Bromocriptine and Cabergoline. She subsequently underwent revision implant augmentation with good outcomes.

**Discussion:**

This case highlights the increased likelihood of post-operative infection in galactorrhoea associated with breast implant augmentation. It is important to exclude lactation preoperatively and avoid a prosthesis in this situation, to minimise this risk and optimise surgical outcomes.

**Conclusion:**

Aesthetic breast surgeons must be aware of the incidence of galactorrhoea, and its possible effects on risks of postoperative complications and poor aesthetic outcomes. The authors suggest deferring implant augmentation until complete resolution of lactation where possible.

## Introduction

Persistent lactation, or galactorrhoea, is a common problem characterised by inappropriate milk production affecting 20–25% of women.^1^ It is most frequently physiological and caused by nipple stimulation and pregnancy; it may also be medication-induced, or more rarely secondary to pituitary prolactinomas.^1^ It is uncommon in the setting of aesthetic surgery, however increasing popularity of procedures such as breast augmentation suggests a need for heightened understanding of the effect of galactorrhoea on surgical outcomes.

Important post-operative outcomes include patient satisfaction, post-operative appearance, and return to normal activities.^2^ Post-operative complications, which mar the success of aesthetic procedures, commonly include capsular contracture, haematoma, and infection, the latter of which complicate approximately 5% of implant augmentations.^3^ Mitigating factors that may worsen surgical outcomes is paramount, and the impact of lactation on outcomes of breast implant augmentation is poorly reported on.

We present the case of a patient with undiagnosed physiological galactorrhoea complicating bilateral breast reduction and implant augmentation. The patient developed a surgical site infection requiring the removal of implants and specialist medical treatment prior to definitive breast surgery.

## Case presentation

**Figure 1:**
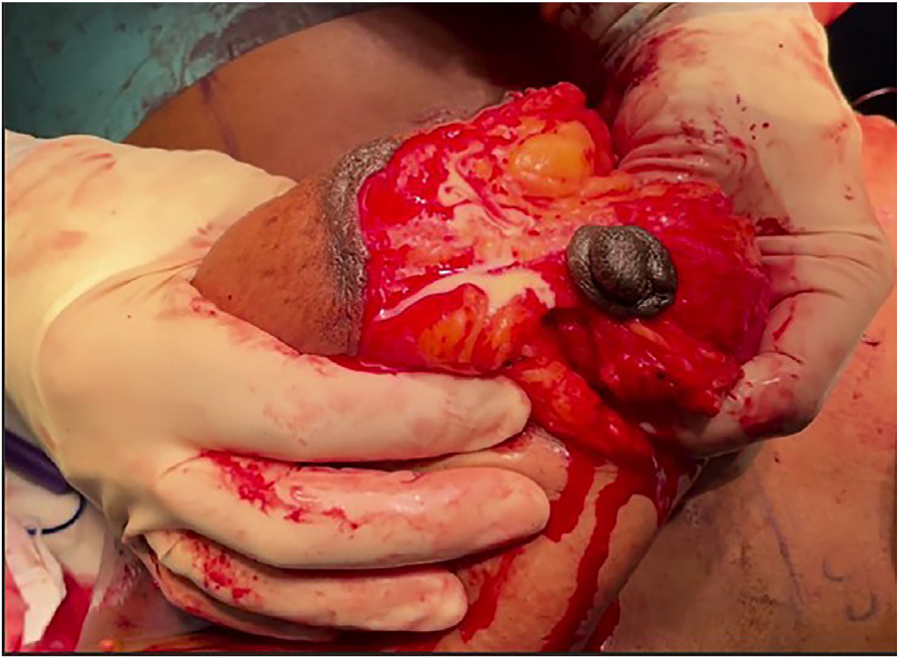
Lactating breast tissue identified intra-operatively.

A 34-year-old patient underwent a ‘mummy makeover’ with bilateral breast implant augmentation and mastopexy, abdominoplasty and bilateral liposuction to the flanks. She had three prior pregnancies, had completed her family, and had stopped breastfeeding over 6 months prior. On initial pre-operative assessment, her breasts were not visibly engorged. The breast reduction was performed via Wise pattern approach based on a superomedial pedicle. Breast tissue was excised from the inferior pole (425 g Left, 477 g Right) and augmented with submuscular (muscle-split technique) 270cc round silicone prostheses.^4^ Intraoperatively, the breast tissue was noted to be lactating (Figure 1). The procedure was completed as planned and a routine postoperative plan was followed including oral antibiotics, analgesia, mobilisation, and compression garment use.

**Figure 2:**
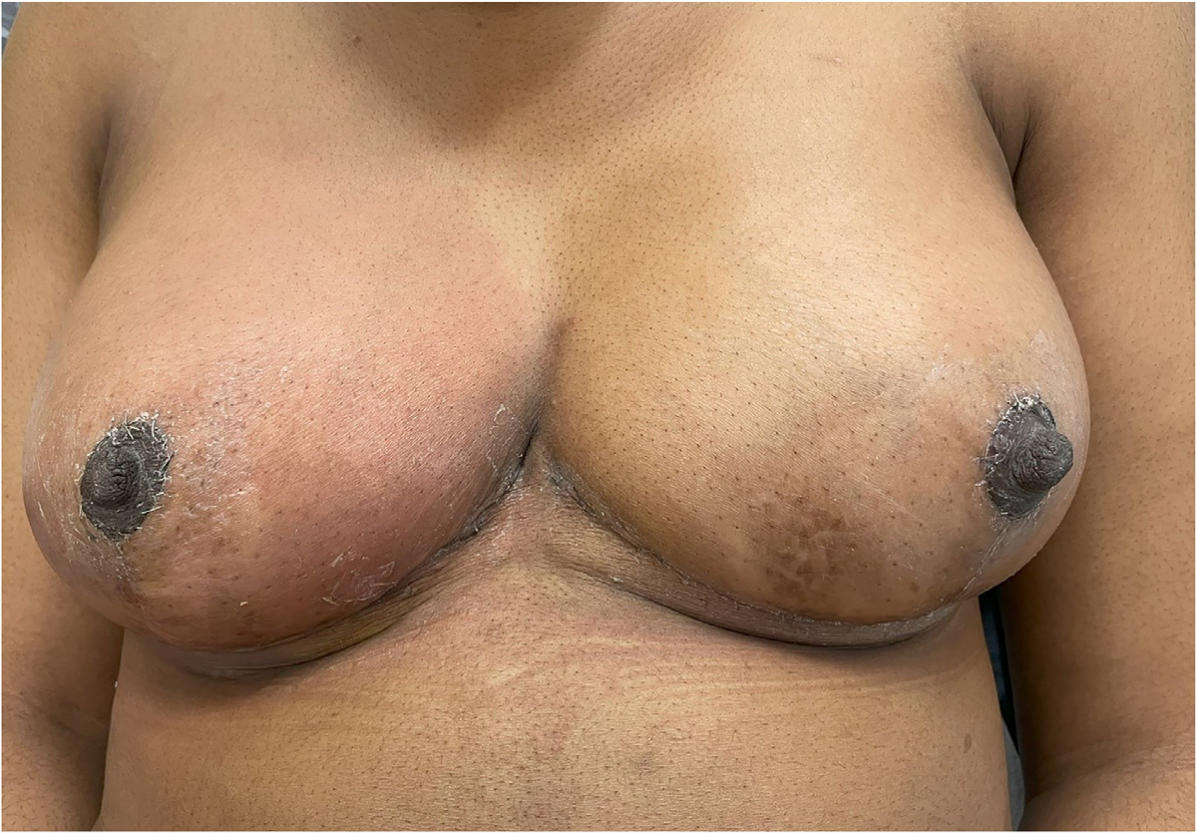
Right sided mastitis on day 10 post-discharge following bilateral implant augmentation and mastopexy.

The patient reattended 10 days later unwell with pain and fevers, her right breast was erythematous and tender (Figure 2). She was diagnosed with a post-operative surgical site infection and admitted for treatment. She underwent removal of bilateral breast implants with washout of significant pus contents from the right breast cavity, antibiotic therapy consisted of gentamicin and co-amoxiclav cover intra-operatively and completion of a 7-day course of oral co-amoxiclav, 625 mg TDS.

**Figure 3a and b:**
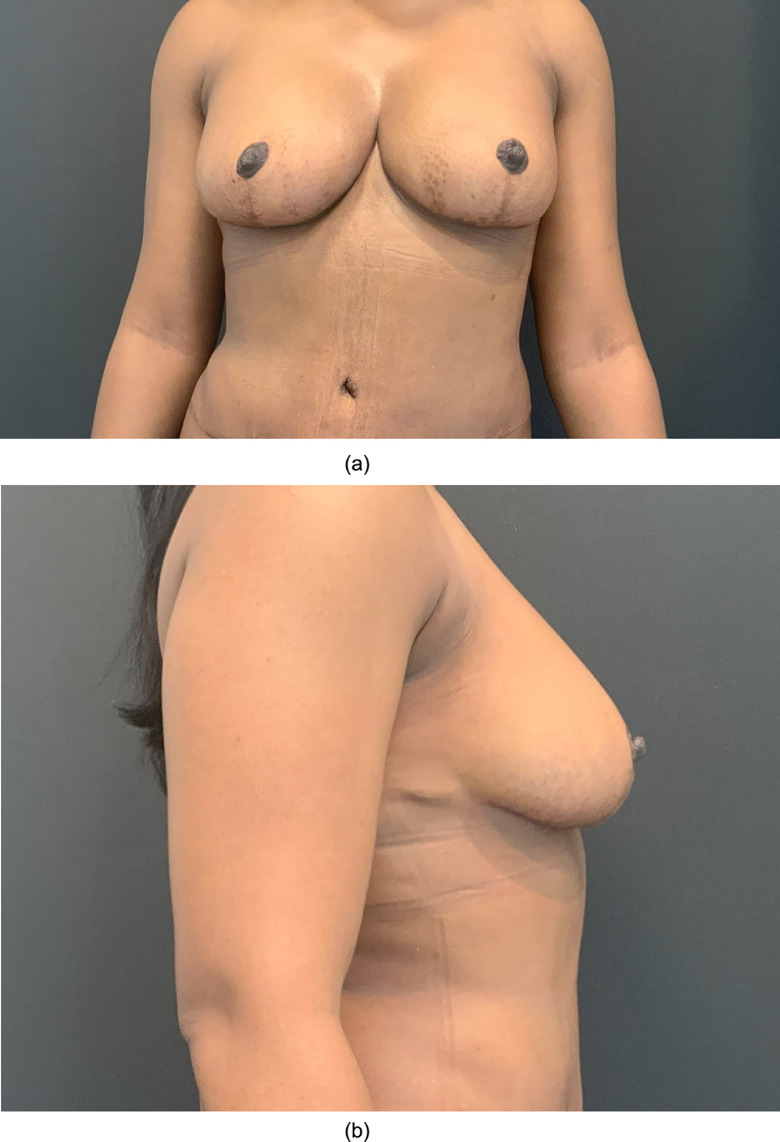
Post-operative result following revision implant augmentation.

She remained well thereafter and was referred to Endocrinology at her local hospital for management of persistent lactation. This eventually resolved after treatment with Bromocriptine and Cabergoline. Upon complete cessation of galactorrhoea, the patient underwent revision implant augmentation 18 months after her initial procedure. She recovered well and was pleased with final outcome (Figure 3).

## Discussion

This case outlines a right breast implant-associated infection, in the context of unexpected physiological galactorrhoea at the time of bilateral breast reduction and implant augmentation. The patient required removal of implant, urgent washout, antibiotic therapy and subsequent endocrine treatment of galactorrhoea, prior to definitive breast augmentation.

Lactation is a well-known risk factor for mastitis, in part due to increased pressure in the breast, leading to oedema and inflammation of surrounding tissues.^5^ Complications relating to lactation in patients with breast implants are poorly described in the literature, but lactation is likely to be associated with infection, which more than often requires explantation for definitive management. Foreign material in the breast has been shown to disrupt immune response and increase susceptibility to bacterial infection, in part due to the formation of a biofilm on the implant.^6^

Kornfeld et al. successfully managed infected breast implants and galactocele in a breastfeeding woman who had had an augmentation 3 years prior to pregnancy. This required incision and drainage of the galactocele, multiple courses of antibiotics, and finally removal of the implants and capsulectomy.^7^ A systematic review by Sharma et al. found 38 women with galactorrhoea following breast implantation, 26% (10/38) of these patients were treated with antibiotics and 21% (8/38) women required implant removal.^3^ A case of galactorrhoea secondary to breast implants was also reported by Suslavicius et al., where the lactation reflex was thought to be triggered by multiple procedures and direct nerve irritation. The case also reported infection, and management consisted of antibiotic therapy with implant removal, with Bromocriptine used to treat her hyperprolactinaemia.^8^

Given the increased risk of infection in galactorrhoea, we suggest implantation of prosthetic material should be contraindicated in this situation. Surgeons should ensure that lactation has resolved prior to surgery; if galactorrhoea is encountered only intraoperatively the temptation to persist with a prosthesis should be resisted. In the case described here, an intraoperative decision to delay the implant augmentation until resolution of lactation may have avoided this postoperative complication requiring urgent readmission to hospital for acute treatment, washout, and explantation procedure. The decision to not proceed with a prosthesis in the lactating breast would minimise the risks of postoperative infection, sepsis and mortality, as well as the associated healthcare costs of an additional operation and admission to hospital.

Furthermore, breast engorgement due to lactation may affect preoperative surgical planning. Acceptable aesthetic outcomes relating to patient satisfaction, postoperative appearances, and return to normal activities require meticulous assessment of the patient's tissue coverage and breast volume, which will fluctuate in a lactating breast.^9^ For aesthetic purposes, the lactating breast is essentially a moving target until galactorrhoea is resolved. Improved aesthetic appearances of the breast are central to outcomes of these procedures; deferring surgery until galactorrhoea is treated will result in improved patient satisfaction and aesthetic outcomes.

Galactorrhoea should be managed by specialist referral to Endocrinology, who will investigate possible causes and commonly instigate dopamine receptor agonist treatment. Whilst galactorrhoea is most commonly of benign aetiology, more sinister causes must be excluded.^1^^,^^3^ In the case presented, no such cause was found, but the galactorrhoea required prolonged treatment for over a year, with multiple dopamine agonists: Bromocriptine and Cabergoline. Upon resolution of lactation, the patient returned for bilateral implant augmentation 18 months after the index procedure. She was satisfied with the outcome of this surgery and has remained complication-free.

## Conclusion

This case describes an unexpected finding of persistent lactation in a patient who underwent bilateral breast implant augmentation and mastopexy. The patient required implant removal and washout. Galactorrhoea may affect surgical planning and increase the risk of post-operative infection; it must be identified pre-operatively to mitigate these risks.

## Compliance with ethical standards

The patient gives their full informed consent to publication of this manuscript.

## Funding

This research did not receive any specific grant from funding agencies in the public, commercial, or not-for-profit sectors.

## Declaration of competing interest

None.

## Appendix Supplementary material

Image, video 1
